# Electrocatalytic PANI-Encapsulated Aluminum Silicate/Ceramic Membranes for Efficient and Energy-Saving Removal of 4-Chlorophenol in Wastewater

**DOI:** 10.3390/membranes15040114

**Published:** 2025-04-07

**Authors:** Shuo Wang, Tianhao Huang, Haoran Ma, Zihan Liu, Houbing Xia, Zhiqiang Sun, Jun Ma, Ying Zhao

**Affiliations:** 1 State Key Laboratory of Urban Water Resource and Environment, Harbin Institute of Technology, Harbin 150090, China; 2 School of Computing, Nankai University, Tianjin 300073, China; 3 School of Marine Science and Technology, Harbin Institute of Technology Weihai, Weihai 264209, China

**Keywords:** electrocatalytic membrane technology, chlorophenol degradation, dechlorination, oxidation

## Abstract

The removal of chlorinated organic pollutants from wastewater is a critical environmental challenge, as traditional methods for treating toxic pollutants like phenol and chlorophenols often suffer from high energy consumption and long treatment times, limiting their practical use. Electrocatalytic filtration has emerged as a promising alternative, but efficient, energy-saving electrocatalytic membranes for pollutants like 4-chlorophenol (4-CP) are still underexplored. A new type of electrocatalytic coupling membrane catalyst, ASP/CM (PANI-encapsulated aluminum silicate/ceramic membrane), was prepared using inexpensive silicate and polyaniline as the base materials, with in situ polymerization combined with co-focus magnetron sputtering. Under optimal conditions (25 mA/cm^2^, 10 mM Na_2_SO_4_, 1.0 mL·min^−1^ flow rate, and 50 μM 4-CP concentration), the membrane achieved about 95.1% removal of 4-CP and the degradation rate after five cycles was higher than 85%. In addition, O_2_^•−^ and •OH are important active species in the electrocatalytic degradation of 4-CP. The 4-CP electrocatalytic membrane filtration process is a dual process of cathode reduction dechlorination and anodic oxidation. This work offers new insights into developing next-generation electrocatalytic membranes and expands the practical applications of electrocatalytic filtration systems.

## 1. Introduction

Chlorinated aromatic compounds, such as 4-chlorophenol (4-CP), are prevalent pollutants in aquatic environments, posing significant risks to human health and ecosystems even at trace concentrations [1,2,3]. Current water treatment technologies often struggle to effectively remove these contaminants, particularly under challenging conditions of low concentrations and high flow rates. Therefore, there is an urgent need to develop innovative processes that are cost-effective, environmentally friendly, safe, and free from secondary pollution to address the 4-CP contamination problem [4,5,6].

Electrochemical oxidation (EO) processes have demonstrated efficacy in removing a wide range of micropollutants, including chlorinated organic compounds, organic dyes, and volatile organic compounds (VOCs) [7,8,9]. However, despite substantial progress in the development of various electrocatalysts, the full potential of EO processes is often constrained by limitations in mass transfer. Recently, an emerging technology—electrochemical membrane reactors (EMRs)—using conductive membranes as flow electrodes has garnered global attention [10,11,12,13]. EMRs integrate membrane separation with electrochemical reactions, enabling simultaneous pollutant removal and electrochemical treatment under an externally modulated electric field. The conductive membrane functions as an electrode, facilitating electrocatalytic reactions that produce reactive species such as hydroxyl radicals and superoxide anions, significantly enhancing pollutant degradation. Furthermore, the electric field promotes contaminant adsorption onto the membrane surface, the generation of secondary reactive species, and a self-cleaning effect by reducing fouling and scaling [14,15]. Meng Sun et al. [16] revealed that electrified membranes (EMs) can overcome the limitations of conventional membrane technologies, providing enhanced performance in water treatment, fouling control, and decontamination. The synthesis of electrocatalysts and their uniform loading onto membranes—considering factors such as nanostructure, particle size, loading amount, and distribution—is crucial for optimizing performance. An efficient and controllable method for electrocatalyst preparation and deposition is essential to ensure even distribution across the membrane surface and within its pore walls, maximizing the porous structure and surface area for effective pollutant degradation. Zishang Chen et al. [17] demonstrated that an electrochemical membrane reactor with full-cell reactions (EMR-P), utilizing a carbon membrane and a TiO_2_-loaded carbon membrane, achieved superior performance in phenol and COD removal. This was attributed to the synergistic effects of electro-Fenton and electrocatalytic oxidation, combined with high adsorption properties. Similarly, Meudjeu Tognia et al. [18] highlighted the untapped potential of modeling and simulation in enhancing the design and performance of membrane–electrode systems integrated with electrocatalytic oxidation processes for treating organic pollutants in water. The primary mechanisms of electrical transport in these systems include (i) the mass transport of micropollutants to the membrane surface, (ii) (electro)adsorption, (iii) direct electron transfer, (iv) the generation of secondary reactive species, and (v) the desorption of by-products. Despite the notable advantages of EMRs, which extend beyond traditional separation functions, these systems are still hindered by structural and surface defects in the membranes. Confocal magnetron co-sputtering technology, with its advantages of high deposition rates, flexible material combinations, and uniform thin films, offers a promising solution to address these challenges [19,20,21].

This work demonstrates the transformative potential of electrochemical membrane reactors (EMRs) as an integrated platform for addressing the dual challenges of efficient degradation and sustainable management of chlorinated aromatic compounds in wastewater. By synergistically coupling membrane filtration with electrocatalytic oxidation, EMRs establish a paradigm-shifting strategy that achieves unprecedented pollutant removal efficiency (>95.1% for 4-chlorophenol within minutes), ultralow energy demand, and operational robustness (five-cycle stability with <5% activity loss), fundamentally overcoming the limitations of conventional technologies in byproduct suppression and process sustainability. The system’s core innovation lies in its spatially resolved reaction mechanism: cathodically generated hydrogen radicals (H*) enable hydrodechlorination through site-specific interactions with adsorbed 4-CP*, while anodic oxidative species synergistically mineralize dechlorinated intermediates, with membrane-confined hydroxyl radicals (•OH) ensuring complete micropollutant elimination. These findings not only decode the atomic-level dechlorination pathway but also validate EMRs as a scalable technology for practical implementation, bridging the critical gap between fundamental electrocatalytic principles and real-world water remediation applications for persistent organic pollutants.

## 2. Materials and Methods

### 2.1. Materials and Chemicals

Information about raw material manufacturers and purity of reactants is summarized in Figure S1.

#### 2.1.1. Synthesis of Polyaniline (PANI)

Polyaniline (PANI) was synthesized via chemical polymerization using potassium persulfate (K_2_S_2_O_8_) as the oxidant. In this study, the molar ratio of oxidant to aniline was set at 1.25. Specifically, 3.7 g of K_2_S_2_O_8_ was dissolved in 75 mL of 0.2 mol·L^−1^ HCl, followed by the dropwise addition of 1 mL of aniline (C_6_H_5_NH_2_, molar mass = 93.13 g·mol^−1^) under constant stirring at room temperature for 12 h. Upon mixing the aniline with the oxidant solution, the reaction mixture turned dark green, indicative of the formation of the emeraldine salt form of PANI. The precipitated PANI powder was collected by filtration, washed with 0.2 mol·L^−1^ HCl until the yellowish filtrate became colorless, and subsequently dried at 80 °C for 12 h [22].

#### 2.1.2. Pretreatment of Aluminum Silicate Powder

The aluminum silicate powder was pretreated by ball milling. A mixture of silicon-aluminum ore powder and zirconium balls, in a mass ratio of 30:1, was subjected to ball milling at a speed of 450 rpm. The milling process involved alternating 1.5 h cycles of forward and reverse rotations.

#### 2.1.3. Synthesis of Polyaniline-Coated Aluminum Silicate (ASP)

The ball-milled aluminum silicate powder was mixed with aniline at molar ratios of aluminum silicate to aniline of 2:1, 2:2, 2:3, and 2:4, respectively. This mixing step was completed prior to the addition of K_2_S_2_O_8_. All subsequent steps remained unchanged. The resulting catalysts were designated as ASP-1, ASP-2, ASP-3, and ASP-4, respectively.

### 2.2. Fabrication and Characterization of ASP/CM

ASP/CM was fabricated by confocal magnetron co-sputtering (AJA International, North Scituate, MA, USA). The deposition thickness of ASP was regulated as ~30 nm on both surfaces of the pristine CM. The microscopic morphologies of the membranes were measured by scanning electron microscopy (SEM, SU8230; Hitachi, Tokyo, Japan), and the distributions of different elements were detected by X-ray energy-dispersive spectroscopy (EDS, XFlash 5060FQ; Bruker, Billerica, MA, USA).

Information about the electro-filtration performance tests, characterization of ASP/CM materials, and DFT calculations is listed in Figures S2–S4.

## 3. Results and Discussion

**Figure 1:**
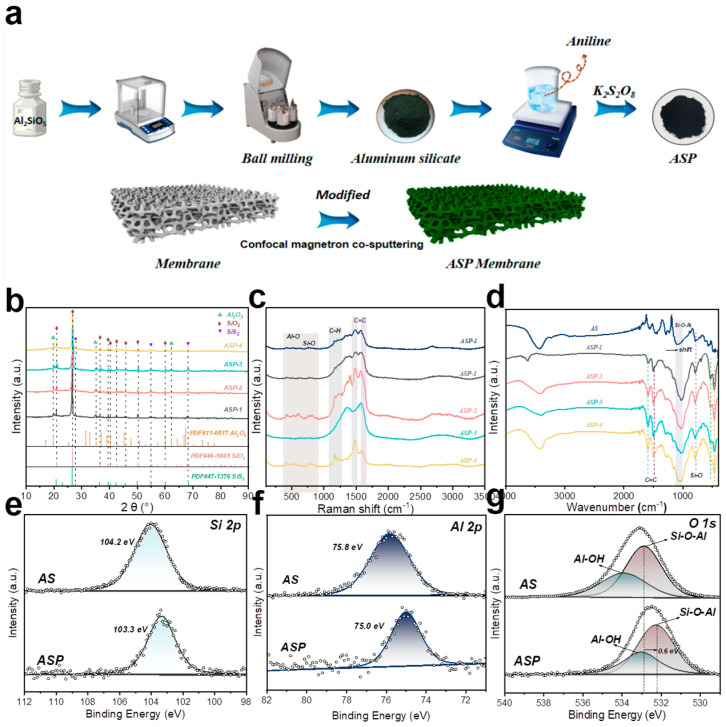
(**a**) Synthesis route of the catalyst. (**b**) XRD pattern of aluminum silicate (AS) and polyaniline-coated aluminum silicate (ASP). (**c**) Raman spectra of all catalysts. (**d**) FT-IR spectra of all catalysts. XPS spectra of catalysts: (**e**) Si 2p; (**f**) Al 2p; and (**g**) O 1s.

**Figure 2:**
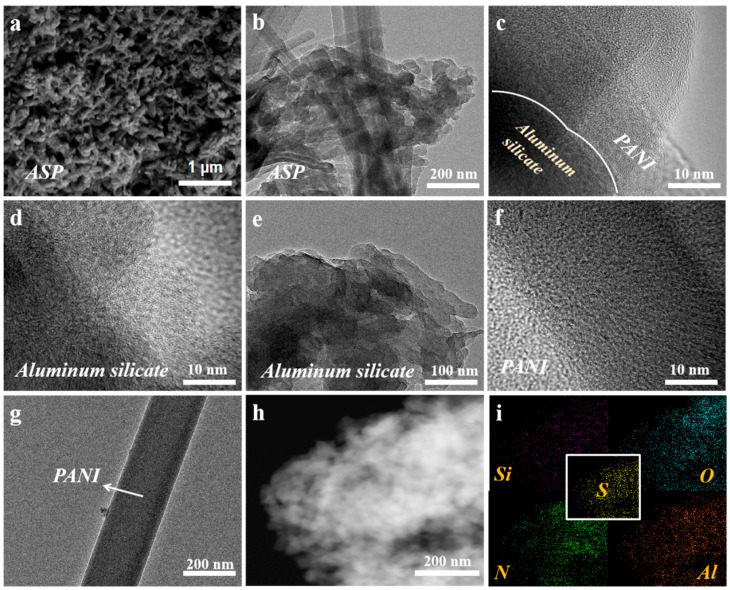
(**a**) SEM image of ASP. (**b**,**c**) TEM images of ASP. (**d**,**e**) TEM images of aluminum silicate. (**f**,**g**) TEM images of PANI. (**h**,**i**) EDX elemental mapping of ASP.

Figure 1a illustrates the preparation method of ASP/CM via in situ polymerization and confocal magnetron co-sputtering. X-ray diffraction analysis (Figure 1b) confirmed that ASP consists of aluminum oxide, silicon oxide, and silicon sulfide (impurities) [23]. The functional groups on the ASP surface were then analyzed using Raman (Figure 1c) and FT-IR spectroscopy (Figure 1d) to investigate the effects of polyaniline introduction. In Figure 1c, characteristic peaks between 500 and 800 cm^−1^ correspond to the stretching vibrations of Si-O and Al-O bonds [24,25]. The addition of PANI significantly enhanced the C=C bond peaks at 1480–1500 cm^−1^ and 1570–1600 cm^−1^ [26]. Notably, the Si-O-Al bond features in the FT-IR spectrum (Figure 1d) shifted to lower frequencies. This red shift is due to PANI’s introduction, which enhances molecular conjugation, reduces the double bond order, and lowers the vibration frequency [27]. The elemental composition and surface chemical states of AS and ASP were further examined using X-ray photoelectron spectroscopy (Figure S1 and Figure 1e–g). Survey spectra revealed clear signals for Si, Al, and O in both AS and ASP [28,29]. The XPS analysis reveals characteristic binding energy signatures at 104.2 eV and 103.3 eV, corresponding to the Si 2p orbital (Figure 1e), while the distinct peaks observed at 75.8 eV and 75.0 eV are attributed to the Al 2p orbital (Figure 1f), reflecting their respective chemical states and coordination environments within the material system. The deconvolution of the O1s XPS spectra reveals pronounced chemical-state evolution, with characteristic binding energy components at 532.8 eV and 531.2 eV corresponding to the surface hydroxyl groups (Al-OH) and bridging oxygen species in Si-O-Al frameworks, respectively, demonstrating dynamic interfacial restructuring of oxygen-containing functional groups during catalytic operation. Compared to pure AS, ASP exhibited a reduced binding energy, indicating that polyaniline introduces electron flow from PANI to aluminum silicate [30,31]. The morphological structure of ASP, PANI, and aluminum silicate was analyzed using SEM (scanning electron microscopy), TEM (transmission electron microscopy), and EDX (energy-dispersive X-ray spectroscopy) elemental mapping (Figure 2). SEM images revealed that the ASP catalyst consists of sheet-like aluminum silicate and rod-like PANI (Figure 2a). TEM images provided a clearer view of the catalyst surface. Aluminum silicate appeared as nanospheres, approximately 20 nm × 30 nm, while PANI exhibited a rod-like structure about 20 nm in width (Figure 2b). A distinct boundary was observed on the ASP surface, indicating the PANI encapsulation of aluminum silicate (Figure 2c). Figure 2d–e show that aluminum silicate is stacked in layers, with a dense and thin single-layer structure. Figure 2f–g show the smooth, rod-like structure of PANI. EDX elemental mappings confirmed a uniform distribution of Si, S, N, Al, and O elements across ASP (Figure 2h–i and Figure S2). BET (Brunauer–Emmett–Teller) analysis further revealed that PANI loading slightly reduced the specific surface area of aluminum silicate (Figure S3). The specific surface area of aluminum silicate is 38.1 m^2^/g, while that of ASP is approximately 34.8 m^2^/g. In conclusion, the successful preparation of the polyaniline-encapsulated aluminum silicate catalyst was validated by XRD, Raman, FT-IR, XPS, SEM, TEM, and BET analyses.

**Figure 3:**
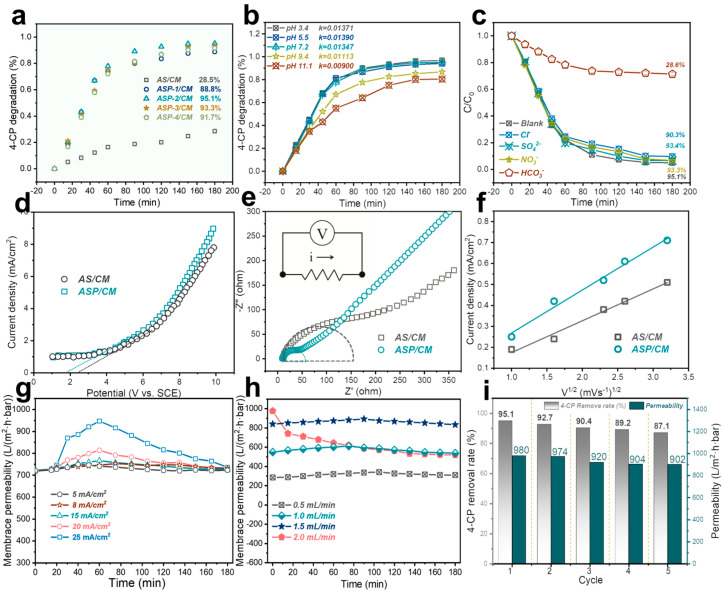
(**a**) Comparison of 4-CP degradation in different systems; (**b**) the influence of pH on 50 μM of 4-CP oxidation; (**c**) effects of Cl^−^, SO_4_^2−^, NO_3_^−^, and HCO_3_^−^ on 4-CP degradation ([Cl^−^]_0_ = [SO_4_^2−^]_0_ = [NO_3_^−^]_0_ = [HCO_3_^−^]_0_ = 10 mM, 10 mM Na_2_SO_4_, 1.0 mL·min^−1^ flow rate, and 50 μM 4-CP concentration, and pH was buffered at 7 with 10 mM phosphate). (**d**) LSV curves of 4-CP oxidation on AS/CM and ASP/CM with 4-CP at 50 mV s^−1^; (**e**) electrochemical impedance spectra (EIS) Nyquist plots of AS/CM and ASP/CM samples using an AC bias of 0 V vs. Ag/AgCl in 0.1 M Na_2_SO_4_ aqueous solution (“i” represents the current, and the arrow indicates the direction of current flow); (**f**) relationship between peak current and square root of scan rate; (**g**) the pure water permeabilities of the pristine membrane under different current densities; (**h**) the pure water permeabilities of the pristine membrane under different flow rates; (**i**) 4-CP removal rates and membrane permeability of ASP/CM cycle filtrations.

To evaluate the catalytic activity of the synthesized AS/CM and ASP/CM composites in the electrocatalytic degradation of 4-CP, the effect of different PANI ratios was systematically analyzed, as shown in Figure 3a. In the absence of PANI, bare AS/CM exhibited a low degradation rate, while all ASP/CM composites showed enhanced electrocatalytic activity, indicating interfacial interactions between ASP/CM and 4-CP in the composites. Notably, the introduction of PANI increased the 4-CP degradation efficiency from 28.5% (AS/CM) to 95.1% (ASP-2/CM). This variation in degradation efficiency is likely due to the different adsorption and binding capacities of the catalysts toward 4-CP, which are influenced by the PANI content.

The effect of pH on 4-CP degradation efficiency was also examined, with the results shown in Figure 3b. The degradation rates of 4-CP decreased rapidly as the pH increased. Figure S4 presents the kinetic model of 4-CP degradation by the ASP-2/CM catalyst under different pH conditions. At pH 3.4, the degradation rate was fastest, reaching 96.8% (R^2^ = 0.995). Acidic pH affects the surface structure of PANI, increasing the -N⁺ content, which enhances the catalyst’s binding ability to 4-CP, thereby improving the degradation efficiency.

As shown in Figure 3c, the impact of various anions (Cl^−^, SO_4_^2−^, NO_3_^−^, HCO_3_^−^) on the electrocatalytic degradation efficiency of 4-CP was investigated. The addition of HCO_3_^−^ ions reduced the degradation efficiency to 28.6%, as HCO_3_^−^ reacts with hydroxyl radicals and shifts the system’s pH to a weakly alkaline range. NO_3_^−^ and SO_4_^2−^ had minimal impact on the system, with the slight decrease in efficiency attributed to competition for active sites. In contrast, the addition of Cl^−^ ions consumed hydroxyl radicals, leading to a decrease in 4-CP degradation efficiency.

Polyaniline, a highly molecularly conductive polymer, enhances electrocatalytic activity, as shown in Figure 3d. Furthermore, electrochemical impedance spectroscopy (EIS) Nyquist plots indicate that coupling PANI improves interfacial charge migration, as evidenced by the reduced semicircle size in the EIS Nyquist plots (Figure 3e). Thus, PANI in the ASP/CM composites serves as an electron reservoir, boosting electron transfer efficiency and improving electrocatalytic activity for 4-CP oxidation. As shown in Figure 3f, a good linear relationship between the peak current and the square root of the scan rate suggests that the mass transfer of 4-CP on the membrane surface is diffusion-controlled. Compared to AS/CM, the physical and chemical properties of the ASP/CM composite membrane provide more favorable confinement for the diffusion of 4-CP.

The efficiency of the electrocatalytic membrane in degrading 4-CP at different current densities was investigated. The optimal current density was found to be 25 mA/cm^2^, selected based on membrane permeability and 4-CP removal rate (Figure 3g and Figure S5). Additionally, the effect of flow rate on degradation efficiency is shown in Figure 3h and Figure S6. The results demonstrated that when the flow rate was below 1.0 mL/min, the extended contact time between the contaminants and the electrocatalytic membrane ensured the complete degradation of 4-CP. However, when the flow rate was increased to 1.5 and 2.0 mL/min, insufficient degradation led to some electrically agglomerated molecules being adsorbed on the membrane surface or in the membrane pores, resulting in decreased permeability. To assess the stability of the ASP-2/CM composite prepared by flow synthesis for the electrocatalytic oxidation of 4-CP, the 4-CP removal rate was monitored for five continuous cycles (Figure 3i). The removal rate remained above 87% throughout, indicating that the ASP-2/CM composite exhibited excellent stability and the potential for long-term, continuous operation.

**Figure 4:**
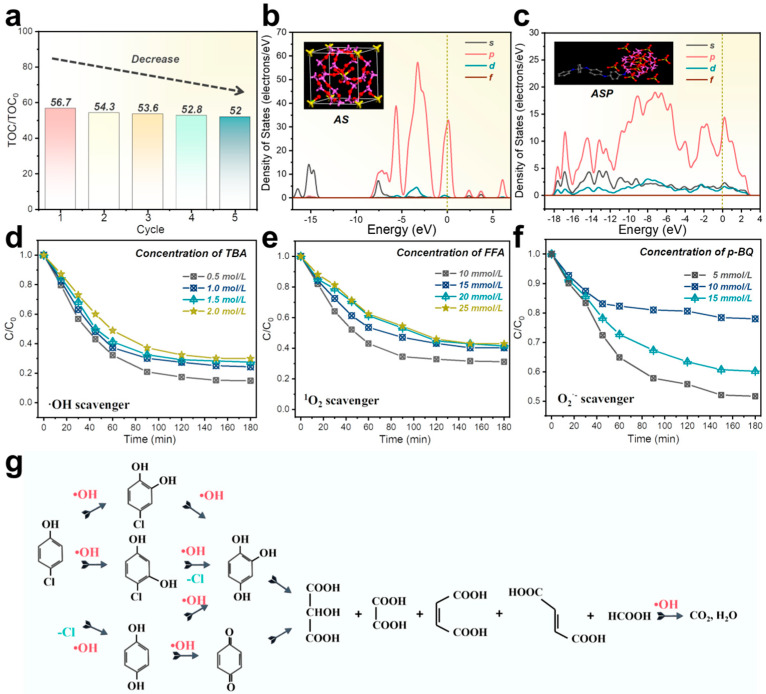
(**a**) TOC removal of 4-CP 5 times. (**b**) Density of states for aluminum silicate (AS). (**c**) Density of states for polyaniline-coated aluminum silicate (ASP). Effects of scavengers on 4-CP degradation in the ASP-2/CM system: (**d**) TBA; (**e**) FFA; and (**f**) p-BQ [10 mM Na_2_SO_4_, 1.0 mL·min^−1^ flow rate, and 50 μM 4-CP concentration; current density =25 mA/cm^2^]. (**g**) Possible degradation pathways of 4-CP.

To evaluate the organic matter removal in the electrocatalytic membrane degradation of 4-CP, the total organic carbon (TOC) value in the water sample was calculated (Figure 4a). The TOC removal efficiency during degradation was approximately 50%, even after five cycles [32,33]. Additionally, the electronic structures of AS and ASP before and after polyaniline doping were analyzed by comparing the density of states (Figure 4b–c). After doping with PANI, the state density of the p orbital significantly increased, the energy range of the density of states (DOS) expanded, and the state density near the Fermi level rose. These changes indicate that PANI doping enhances the material’s conductivity, making it more suitable for electron transfer applications [34,35].

The main active species in the electrocatalytic membrane oxidation of 4-CP were analyzed through various quenching experiments (Figure 4d–f). The degradation efficiency of 4-CP decreased progressively with increasing concentrations of TBA (tert-butanol), with inhibition plateauing at higher concentrations. This suggests that hydroxyl radicals (•OH) play a dominant role in the degradation of 4-CP, and their activity is significantly suppressed by excess TBA (Figure 4d). As the concentration of FFA (furfuryl alcohol) increased, the degradation efficiency of 4-CP also declined, though less markedly than with the •OH scavenger. This indicates that singlet oxygen (^1^O_2_) also contributes to the degradation, albeit secondarily to •OH (Figure 4e). The addition of p-BQ (para-benzoquinone) caused a substantial reduction in 4-CP degradation efficiency, with a more pronounced inhibitory effect than the other two scavengers. This suggests that superoxide anion radicals (O_2_^•−^) play a key role, possibly serving as the dominant reactive species in the process (Figure 4f). Comparing the effects of the three scavengers, it is evident that O_2_^•−^ and •OH are the primary reactive species, while ^1^O_2_ acts as an auxiliary species. The stronger inhibitory effect observed with increasing scavenger concentrations underscores the critical role of these reactive oxygen species in the electrocatalytic oxidation process.

As shown in Figure 4g, in the initial stage of the reaction, 4-CP diffuses to the electrode surface, where it adsorbs and interacts with the hydroxyl radicals produced on the electrode. The degradation products still exhibit a conjugated system or double bond structure similar to the benzene ring, with the ring itself remaining intact. This process is rapid. Intermediate products may include quinone compounds, 2-chlorocatechol, 3-chlorocatechol, and 2-hydroxy-1,4-benzenediol. As the reaction progresses, benzene ring-containing intermediates react with hydroxyl radicals, undergoing ring-opening degradation into organic acids like maleic acid and oxalic acid, a relatively slower process. Some organic acids are further oxidized, ultimately transforming into the final products, carbon dioxide, and water [36,37,38,39,40]. Liquid chromatography analysis also confirmed that the 4-CP concentration gradually decreased over time, consistent with the HPLC-MS results (Figure S7).

### Mechanism Analysis

**Figure 5:**
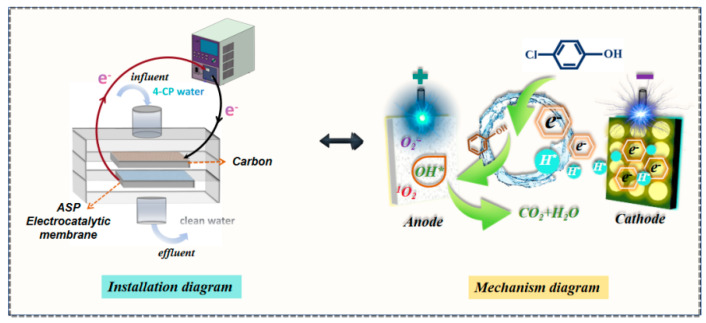
Schematic diagram (**left**) and mechanism diagram (**right**) of electrocatalytic membrane degradation of 4-CP.

This work presents an ASP/CM composite for high-performance 4-CP degradation in a single-pass, flow-through electro-filtration process, as illustrated in Figure 5. The ASP/CM exhibited excellent membrane permeability and hydrophilicity, comparable to the pristine CM substrate. Electrochemical impedance spectroscopy and LSV curve analyses further revealed that the introduction of PANI significantly enhanced charge transfer within the membrane. As a result, the ASP/CM showed a substantial decrease in 4-CP concentration after electrocatalytic membrane filtration. During the degradation process, 4-CP undergoes reduction and dechlorination at the cathode, while the final oxidation products at the anode are carbon dioxide and water. Hydroxyl radicals, singlet oxygen, and superoxide anion radicals all play crucial roles in the degradation of 4-CP, with each species contributing to different stages of the reaction.

H* + Cl-C_6_H_5_O→HCl + C_6_H_5_O

C_6_H_5_O + ROS→CO_2_ + H_2_O

## 4. Conclusions

In this work, a high-performance electrocatalytic microfiltration ASP/CM membrane was prepared by in situ polymerization coupled with confocal magnetron co-sputtering. The introduction of PANI can increase the electron transfer rate at the electrocatalytic interface, enhance the conductivity, and thus improve the degradation and absorption performance of 4-CP. After 3 h of electrocatalytic membrane filtration to degrade 4-CP, the removal rate of 50 μM 4-CP reached 95.1%, while the TOC removal rate in the solution was about 50%. O_2_^•−^ and •OH are important active species in the electrocatalytic degradation of 4-CP. This high-performance electro-filtration system has promising prospects in applications such as fast water purification and water pollution control.

## Data Availability

We have provided all the data supporting the reported results.

## Supplementary Materials

The following supporting information can be downloaded at: https://www.mdpi.com/article/10.3390/membranes15040114/s1, Text S1: Chemicals; Text S2: Electro-filtration performance tests; Text S3: Characterization of ASP/CM materials; Text S4: DFT calculations, Figure S1: XPS spectra of AS and ASP (Survey); Figure S2: Map sum spectrum of ASP, Figure S3: Surface area of AS and ASP; Figure S4: The removal rates of 4-CP at different current densities; Figure S5: The removal rates of 4-CP at different current densities; Figure S6: The removal rates of 4-CP at different flow rate; Figure S7: High performance liquid chromatography of solution during the process of electrochemical degradation of 4-CP at various degradation time.
