# Supplementary material for: Electrocatalytic PANI-Encapsulated Aluminum Silicate/Ceramic Membranes for Efficient and Energy-Saving Removal of 4-Chlorophenol in Wastewater
Source: Membranes (Basel). 2025 Apr 7;15(4):114. doi: 10.3390/membranes15040114 (PMC12028994; doi:10.3390/membranes15040114)
Supplement: Supplementary file 1 [file membranes-15-00114-s001.zip › membranes-3479690-supplementary.pdf]

Support Information for

Electrocatalytic ASP/CM Membranes for Efficient and Energy-Saving Removal of 4-Chlorophenol in Wastewater

Text S1. Chemicals

Aniline (Sigma-Aldrich, St. Louis, MO, USA), potassium peroxydisulfate ($K_2S_2O_8$, Sigma-Aldrich, St. Louis, MO, USA), and aluminum silicate (AS, Sigma-Aldrich, St. Louis, MO, USA) were purchased from Sigma-Aldrich Co. Ltd. Sodium sulfate (Na_2SO_4, J.T.Baker, Phillipsburg, NJ, USA) and ethanol (EtOH, J.T.Baker, Phillipsburg, NJ, USA) were provided by J.T.Baker Chemicals. Pristine ceramic membrane (CM, molecular weight cut-off = 300 kDa, diameter = 47 mm, thickness = 2.5 mm; Sterlitech, Kent, WA, USA) was purchased from Sterlitech Co., composed of titanium oxide (TiO_2) and zirconium oxide (ZrO_2). Aluminum silicate (Aladdin, Shanghai, China), 4-chlorophenol (4-CP, Aladdin, Shanghai, China), furfuryl alcohol (FFA, Aladdin, Shanghai, China), and benzoquinone (BQ, Aladdin, Shanghai, China) were purchased from Aladdin Co. Ltd. Ethanol (Fisher Chemical, Waltham, MA, USA), hydrochloric acid (Fisher Chemical, Waltham, MA, USA), and $K_2S_2O_8$ (Fisher Chemical, Waltham, MA, USA) were obtained from Fisher Co. Ltd. COD analysis reagents (HACH, Loveland, CO, USA) and TOC analysis reagents (HACH, Loveland, CO, USA) were purchased from HACH Inc. Deionized (DI) water was purified using a Milli-Q ultrapure water system (Merck Millipore, Burlington, MA, USA).

Text S2. Electro-filtration performance tests

To evaluate the conductivity enhancement of ASP/CM relative to the pristine CM, electrochemical impedance spectroscopy (EIS) was carried out using an electrochemical workstation (CHI 660E, CH Instruments). The electrochemical workstation used a three-electrode cell with a membrane, a platinum wire, and an Ag/AgCl electrode as the working electrode, counter electrode, and reference electrode, respectively. EIS was tested under the following condition: frequency range

of 10^{-6} Hz, electrolyte solution of 10 mM Na_2SO_4 solution. To investigate the electron-transfer mediating action of ASP/CM, cyclic voltammetry (CV) and chronoamperometry (CA) tests were conducted using the electrochemical working station (PARSTAT302N, Metrohm). During the CV and CA tests, 1/2 piece of the ASP/CM was employed for detection. The two surfaces of the ASP/CM were used as the working electrode and counter electrode, respectively, and an Ag/AgCl electrode was used as the reference electrode. CV measurement was recorded with a scan rate of 100 mV s^{-1}. CA measurement was conducted with the working electrode biased at a given potential.

Text S3. Characterization of ASP/CM materials

The crystal structures of the samples were characterized by X-ray diffraction (XRD, Rigaku, Japan) with the Cu-Ka radiation (λ = 0.15406 nm). The FT-IR spectra (FT-IR, Spectrum 100, USA) were recorded in the range of 400-4000 cm^{-1} by the KBr pellet method. Raman spectra were obtained on a confocal micro-Raman spectrometer (XploRA, France) with an excitation of 532 nm laser light. The specific surface areas were measured by the N_2 adsorption/desorption isotherm (Tristar II 3020, USA) using the Brunauer-Emmett-Teller (BET) analysis method. Elemental analyses were performed by ICP-OES with an ICP spectroflamme-D. TEM images were obtained by a JEM 1200EX transmission electron microscope operated at an accelerating voltage of 100 kV and by a TecnaiTF20 transmission electron microscope. X-ray photoelectron spectra (XPS, XSAM 800, UK) analysis was performed with K-Alpha using an Al Kα (1361 eV).

The 4-CP concentration was determined by High Performance Liquid Chromatography (HPLC) (Shanghai WUFENG, EX1600, China). The HPLC method for 4-chlorophenol (4-CP) determination utilizes a reversed-phase C18 column (e.g.,

250 mm × 4.6 mm, 5 μm) with a mobile phase consisting of methanol/water (60:40, v/v) at a flow rate of 1.0 mL/min, coupled with UV detection at 280 nm for optimal sensitivity; the injection volume is typically 20 μL, and the column temperature is maintained at 30°C, ensuring baseline separation of 4-CP from potential interferences within a retention time of approximately 6-8 min, while calibration curves are constructed using standard solutions in the concentration range of 0.1-50 mg/L to achieve accurate quantification. Total organic carbon (TOC) was measured by a HACH DRB200 thermo-reactor and a HACH DR6000 UV-Vis spectrometer (HACH Inc., USA). The membrane permeability was determined by the following equation:

$$J = \frac{V \times 3600}{t \times \Delta P \times S \times 10000}$$

where J is the membrane permeability (L/(m^2·h·bar)), V is the collected volume of permeate (mL), t is the permeation time (s), S is the available membrane area (m^2) and ΔP denotes the trans-membrane pressure (bar). The 4-CP removal efficiency, TOC removal efficiency were calculated by the following equation:

$$R = \frac{C_f - C_p}{C_f} \times 100\%$$

where R is the 4-CP, TOC removal efficiency (%), C_f is the 4-CP concentration, TOC of feed (mg/L) and C_p is the 4-CP concentration, TOC in the treated sample (mg/L).

Text S4. DFT calculations

The DFT is vital for the interface engineering of photocatalyst with pollutant, which combines the experimental results conducted with the insights mechanism of interface tailoring and guides the interface material design or the trend of interface modification. PANI and aluminum silicate geometric optimizations and single point energy calculations were performed using the

Vienna Ab initio Simulation Package (VASP). The generalized gradient approximation (GGA) with the Perdew-Burke-Ernzerhof (PBE) exchange-correlation functional was chosen to describe the exchange-correlation interactions, and the Castep and DFTB method were chosen to correctly describe van der Waals (vdW) interactions. The plane wave cut-off energies of PANI and aluminum silicate are 450 eV, respectively. The geometric structure was optimized by cubic cells with a side preliminary length of 30 Å, and the Brillouin zone was sampled using only the Γ point, until the convergence criteria of energy and force were less than 10^4 eV and 0.04 eV $Å^{-1}$, respectively.

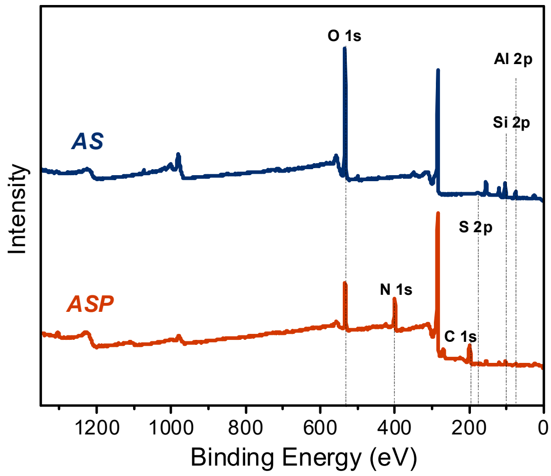

Figure S1. XPS spectra of AS and ASP (Survey).

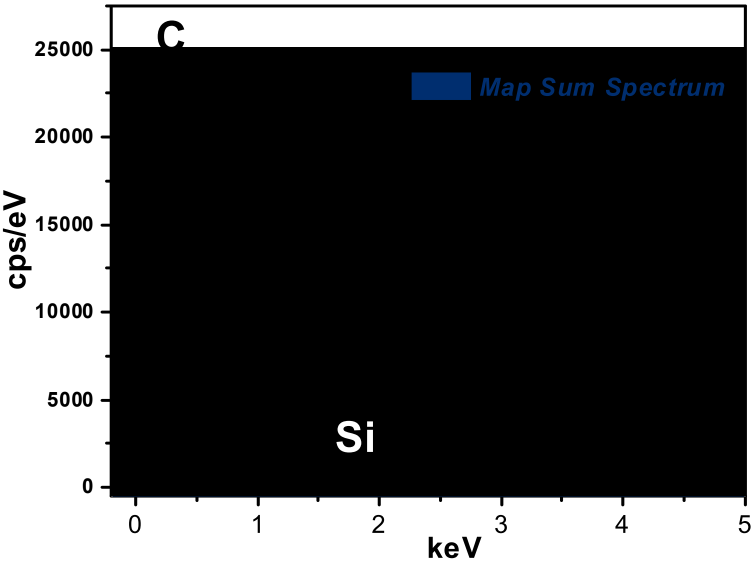

Figure S2. Map sum spectrum of ASP.

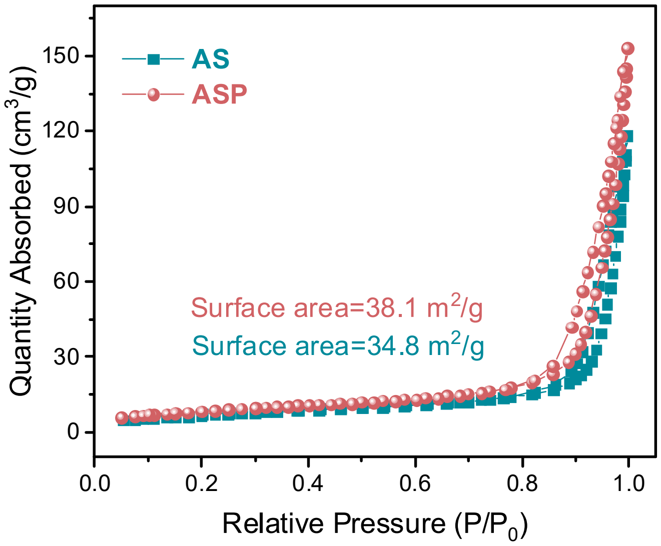

Figure S3. Surface area of AS and ASP.

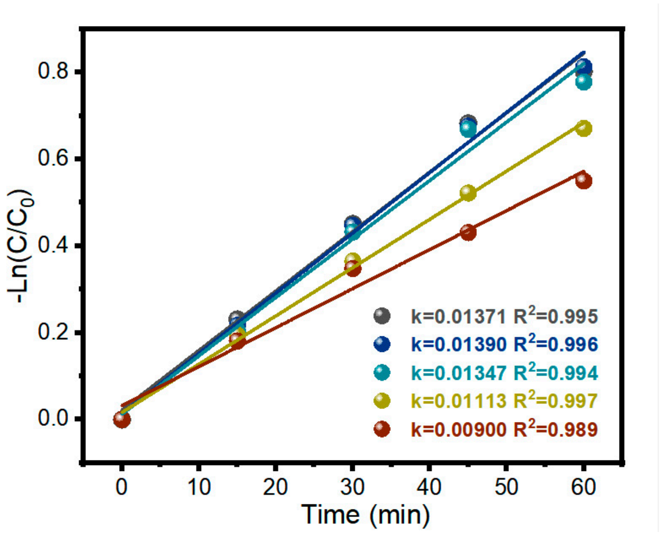

Figure S4. The removal rates of 4-CP at different current densities.

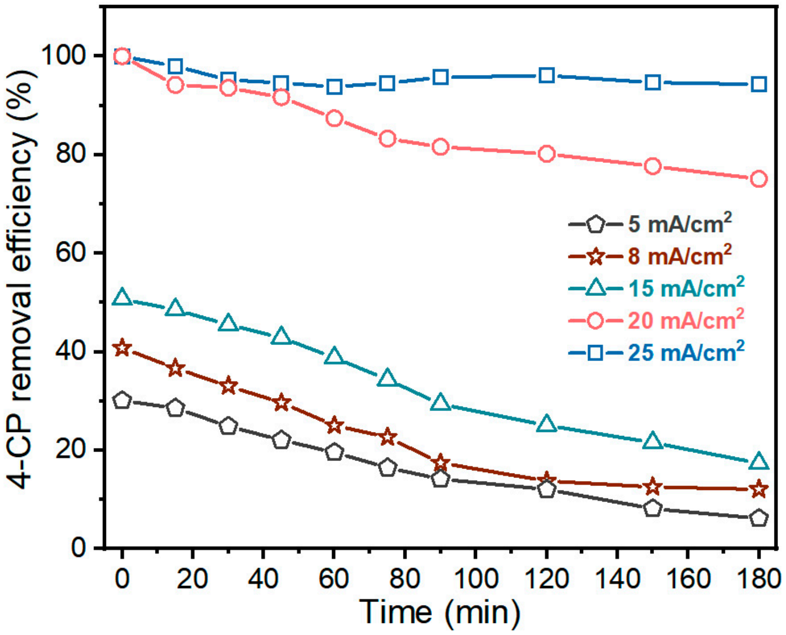

Figure S5. The removal rates of 4-CP at different current densities.

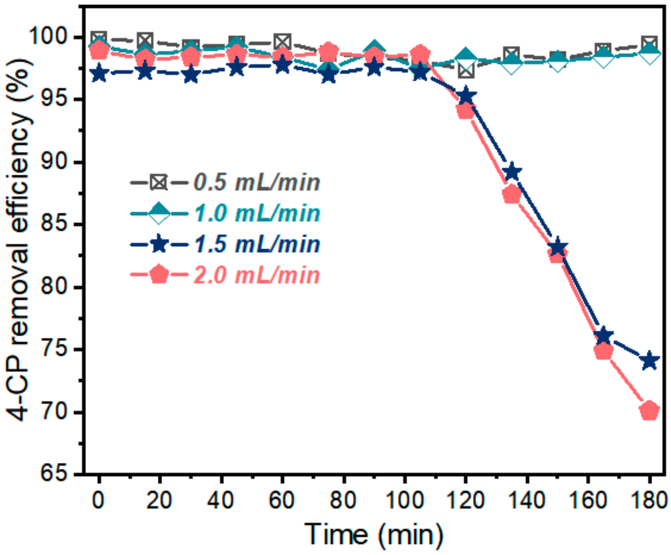

Figure S6. The removal rates of 4-CP at different flow rate.

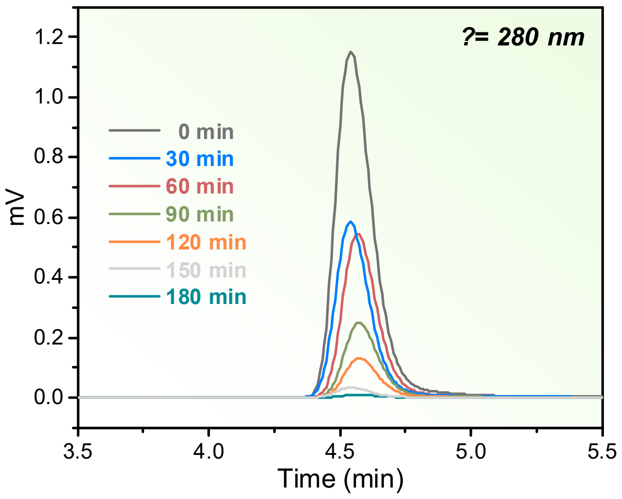

Figure S7 High performance liquid chromatography of solution during the process of electrochemical degradation of 4-CP at various degradation time.
